# Automatic Pancreatic Cyst Lesion Segmentation on EUS Images Using a Deep-Learning Approach

**DOI:** 10.3390/s22010245

**Published:** 2021-12-30

**Authors:** Seok Oh, Young-Jae Kim, Young-Taek Park, Kwang-Gi Kim

**Affiliations:** 1 Gil Medical Center, Department of Biomedical Engineering, Gachon University College of Medicine, Incheon 21565, Korea; seogo1216@gmail.com (S.O.); youngjae@gachon.ac.kr (Y.-J.K.); 2 HIRA Research Institute, Health Insurance Review & Assessment Service (HIRA), Wonju-si 26465, Korea; pyt0601@hira.or.kr

**Keywords:** pancreatic cyst lesion, segmentation, computer-aided diagnosis, deep learning, endoscopic ultrasonography

## Abstract

The automatic segmentation of the pancreatic cyst lesion (PCL) is essential for the automated diagnosis of pancreatic cyst lesions on endoscopic ultrasonography (EUS) images. In this study, we proposed a deep-learning approach for PCL segmentation on EUS images. We employed the Attention U-Net model for automatic PCL segmentation. The Attention U-Net was compared with the Basic U-Net, Residual U-Net, and U-Net++ models. The Attention U-Net showed a better dice similarity coefficient (DSC) and intersection over union (IoU) scores than the other models on the internal test. Although the Basic U-Net showed a higher DSC and IoU scores on the external test than the Attention U-Net, there was no statistically significant difference. On the internal test of the cross-over study, the Attention U-Net showed the highest DSC and IoU scores. However, there was no significant difference between the Attention U-Net and Residual U-Net or between the Attention U-Net and U-Net++. On the external test of the cross-over study, all models showed no significant difference from each other. To the best of our knowledge, this is the first study implementing segmentation of PCL on EUS images using a deep-learning approach. Our experimental results show that a deep-learning approach can be applied successfully for PCL segmentation on EUS images.

## 1. Introduction

The pancreas is located behind the stomach and regulates blood sugar levels by secreting related hormones to the digestive system. A pancreatic cyst lesion (PCL) is an abnormal inflammatory or proliferative lesion of the pancreas [1]. There are not only benign tumors in PCL but also some subtypes of PCL, such as IPMN (Intraductal papillary mucinous neoplasm) and MCN (Mucinous cystic neoplasm), can become malignant tumors [2]. The 5-year survival rate of people with pancreatic cancer is generally 3–15% [3,4]. Therefore, early and precise diagnosis of PCL is important.

In the clinical environment, PCLs are diagnosed by the visual inspection of images recorded from computed tomography (CT), magnetic resonance imaging (MRI), or endoscopic ultrasonography (EUS). Among these imaging modalities, EUS provides a high spatial resolution image and enables repeated procedures such as non-ionizing imaging. In addition, the accuracy of MRI and CT in diagnosing PCLs has been reported at 39–50% and 40–44%, respectively [5,6]. The diagnostic accuracy of EUS for malignant or premalignant PCLs is approximately 95% [6,7]. However, operators of EUS procedures require professional experience and high technical abilities. To acquire comprehensive competence in all aspects of EUS, it is suggested that clinicians perform at least 150 supervised cases [8]. Therefore, the insufficient experience of operators may lead to misdiagnosis, and even an experienced expert can be affected by fatigue and carelessness due to long-term EUS procedures.

Recently, with advances in digital imaging processing and artificial intelligence, a computer-aided diagnosis system (CAD) was developed to assist clinicians in the interpretation of medical images. One of the purposes of the CAD system is to identify the region of interest (ROI) in the image. The detection of segmentation in the lesions automatically separates the ROIs from other areas in medical images. Using the segmented images, a diagnosis is executed by interpreting the characteristics of the ROI. There are reports on the diagnosis of pancreatic lesions using EUS images and traditional approaches [9,10,11,12]; however, they required a manual ROI segmentation process, which is time-consuming. Automatic segmentation without a manual approach is necessary to develop a fully automatic CAD system. There are also a few studies of CAD systems using deep-learning approaches for pancreatic lesions on EUS images.

Nguon et al. developed a deep learning-based CAD system for the differentiation of subtypes of PCL, which are MCN and SCN (Serous cystic neoplasm) [13]. They achieved an accuracy of up to 82.75% and an area under the receiver operating characteristic (AUROC) score of 0.88. However, they also conducted a manual ROI segmentation process.

Iwasa et al. studied deep-learning-based automatic segmentation of pancreatic tumors on contrast-enhanced EUS (CE-EUS) [14]. They extracted images from CE-EUS videos with six frames per second. They achieved a median intersection over union (IoU) of 0.77 in all cases. Furthermore, they divided the dataset according to the tumor boundary of visibility. The median IoU of TB-1 (tumor boundary of visibility all around) was 0.80, TB-2 (tumor boundary of visibility 50–99% around) was 0.76, and TB-3 (tumor boundary of visibility less than 50% around) was 0.69. However, they did not consider the segmentation of cystic lesions. Therefore, it is not clear whether cystic lesions of the pancreas can be effectively segmented using deep-learning approaches.

Zhang et al. used deep-learning algorithms for the detection of pancreas location and recognition of EUS station [15]. However, they conducted pancreas segmentation related to anatomical structures, such as the abdominal aorta, pancreatic body, pancreatic tail, confluence, pancreatic head from the stomach, and pancreatic head from the descending part of the duodenum; they did not study pathological lesions like PCL.

Tonozuka et al. developed a deep learning-based CAD system for pancreatic cancer detection using a 7-layer convolutional neural network (CNN) from a single-institution dataset [16]. They achieved an AUROC score of 92.4% for the validation set and 94.0% for the test set. Lesion detection is able to build only a rectangular- or square-bounded box. However, lesion segmentation provides a pixel-wise mask for identifying lesion shapes. Therefore, the segmentation process provides valuable information that can be used to analyze PCLs.

Although several studies have dealt with pancreatic lesion detection or classification on EUS images and one study developed deep learning-based pancreatic tumor segmentation, PCL segmentation using a deep-learning approach has rarely been studied. This study implemented automatic PCL segmentation on EUS images based on a deep-learning approach.

## 2. Materials and Methods

### 2.1. Data Used and Preprocessing

For dataset A, the EUS data of 52 patients (57 images) were collected from the Gil Medical Center (IRB number: GDIRB2020-317). For algorithmic evaluation, all data were segmented manually by one experienced radiologist. Moreover, the EUS data of 59 patients (364 images) collected from the Severance Hospital was used for dataset B.

We implemented five-fold cross-validation to train the deep-learning model. In these steps, we used EUS data from 39 patients (43 images) to develop the model. For the internal test, EUS data from 13 patients (14 images) were used. Moreover, we performed the external test on all of dataset B (59 patients, 364 images) by using the models developed on the training data (39 patients, 43 images) from dataset A. Table 1 shows the dataset description.

Additionally, we conducted a cross-over study. For the cross-over study, we developed the models on the 46 patient’s EUS data (288 images) of dataset B. For the internal test on the cross-over study, we evaluated the models on the 13 patient’s EUS data (76 images) of dataset B. We executed the external test on all of dataset A (52 patients, 57 images) by using the models developed on the training data (46 patients, 288 images) from dataset B.

**Figure 1:**
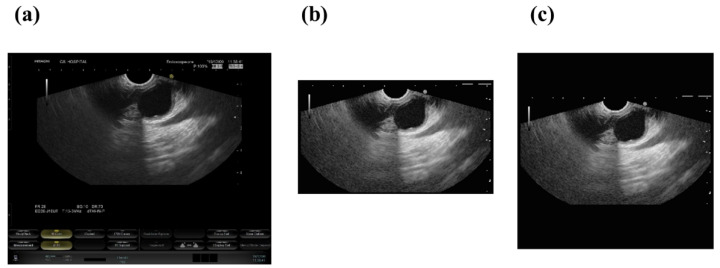
Image processing and zero-padding of the endoscopic ultrasonography images. (**a**) Original image, (**b**) Histogram equalized and cropped image, and (**c**) padded image.

We pre-processed the EUS images on dataset A and dataset B. We cropped each EUS image using a rectangular box to eliminate unnecessary areas, including endoscopic images, manual bars, and other irrelevant spaces. These cropped images were converted to grayscale images. Next, adaptive histogram equalization was applied to the image for contrast enhancement. The cropped images were zero-padded to 1024 × 1024 pixels; however, due to a limited computing capacity, the images were resized to 256 × 256. Figure 1 shows the original EUS images and the pre-processed images. We implemented data augmentation based on a geometric transformation to avoid overfitting by using uniform scaling (0.5, 1.5), rotation (−22.5°, 22.5°), and vertical and horizontal translation (−32 pixels, 32 pixels). Finally, the EUS images were normalized to the (0, 1) scale. The augmentation was randomly performed 100 times when training the models on dataset A and 20 times when training the models on dataset B.

### 2.2. Deep Learning-Based Segmentation

**Figure 2:**
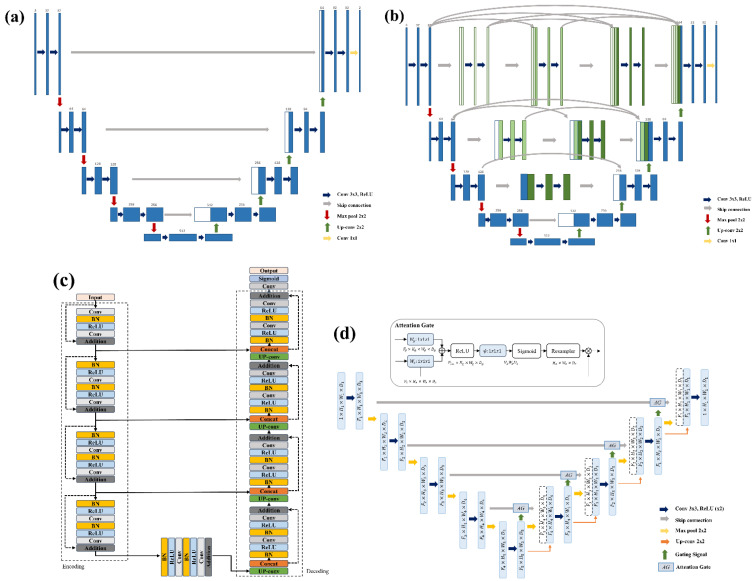
Architecture of each model, (**a**) Basic U-Net, (**b**) U-Net++, (**c**) Residual U-Net, and (**d**) Attention U-Net.

Currently, the most common algorithm for deep learning-based segmentation is the U-Net architecture [17]. The U-Net [18] is a convolutional network architecture for the fast and precise segmentation of medical images. The U-Net architecture is composed of an encoder and a decoder. The encoder captures the context information, and the decoder precisely localizes the captured information on the image. Several variants of the U-Net model can improve the performance of medical image segmentation. These variants were developed by incorporating other deep-learning techniques. In this study, we employed the Attention U-Net [19] for automatic PCL segmentation. Each level of the decoder in the Attention U-Net includes an attention gate. The attention gate [20] focuses on important regions and ignores irrelevant regions in the image. Feature maps from the corresponding encoder were passed through an attention gate to highlight valuable features. Subsequently, the outputs of the attention gate were concatenated with up-sampled feature maps from the low-level layer. We also employed the Residual U-Net [21], U-Net++ [22], and Basic U-Net for comparison with the Attention U-Net. Figure 2 represents the deep-learning architectures used in this study.

The encoder of U-Net-based models is composed of several convolution blocks including two 3 × 3 convolution layers, ReLU (Rectified Linear Unit) activation function, and the max-pooling layer. In the decoder, feature maps are up-sampled by a 2 × 2 convolution layer and are concatenated with corresponding feature maps in the encoder by skip-connection. Next, two 3 × 3 convolution layers and ReLU activation functions follow. Finally, a 1 × 1 convolution layer reduces the feature maps and produces the segmentation output.

The Residual U-Net is based on the residual network [23]. The residual block is designed to prevent the gradient from vanishing and exploding in a deeper network and has a skip connection that adds output from the previous block to the output of the next block. The Residual U-Net applies the summation between input feature maps and output feature maps in each convolution block. Therefore, this model aids the preservation of the meaningful information of the feature map and improves the segmentation performance in a deeper network without any gradient vanishing and exploding.

The U-Net++ is one of the powerful U-Net variants for medical image segmentation. In this model, dense skip-connection inspired from densely connected convolutional networks [24] is used to connect the corresponding encoder and decoder layer through a series of nests. The U-Net++ has a number of skip-connections at each level. Each skip connection takes the feature maps from the previous block and the up-sampled feature maps from the low-level block. This architecture enables the reduction of the semantic gap between the feature maps of the encoder and decoder.

### 2.3. Implementation

Deep learning-based segmentation algorithms were trained under equivalent conditions using a 5-level encoder and decoder with channels of 32, 64, 128, 256, and 512 in the convolution layer. We initialized the weights in a normal distribution and trained all networks from scratch. We used the Adam optimizer [25] with a mini-batch size of eight. The binary cross entropy (BCE)–Dice loss, which combines the Dice loss [26] with the binary cross-entropy loss [27], was used for the loss function. The learning rate was initialized at 0.0001 and was divided by two when the error plateaued. The training was stopped as soon as validation loss was not decreasing in 20 epochs.

### 2.4. Evaluation Metric

We evaluated the segmented mask by comparing the positive pixels between the ground truth and the prediction. Two quantitative analysis metrics were considered: the dice similarity coefficient (DSC) and IoU. The DSC is twice the overlap area between the ground truth and the predicted positive pixels, divided by the total number of positive pixels of both the ground truth and prediction. The IoU is the area of overlap between the ground truth and the predicted positive pixels, divided by a union between the ground truth and prediction. We calculated the pixel accuracy, pixel specificity, and pixel sensitivity to evaluate the pixels that were segmented correctly in the image. Moreover, we calculated the recall score, which was the number of images with an IoU score greater than a predefined threshold, divided by the total number of images. The thresholds were set to 0.5, 0.7, and 0.85. Furthermore, the Recall–IoU curve was used to evaluate the average performance at different levels.

## 3. Results

The deep learning-based PCL segmentation results are presented in this section. We trained the models using 5-fold cross-validation on the training data of dataset A (39 patients, 43 images). We selected the final model on the 5-fold cross-validation that showed the lowest validation loss. The final model was used for the blindfold test. The internal test was executed on the test data of dataset A (13 patients, 14 images). The external test was executed on all of dataset B (59 patients, 364 images).

Moreover, we performed a cross-over study that developed the model on the large dataset and evaluated the model on the small dataset. On the cross-over study, we trained the models using 5-fold cross-validation on the training data of dataset B (46 patients, 288 images). We selected the final model on the 5-fold cross-validation that showed the lowest validation loss. The final model was used for the blindfold test on the cross-over study. The internal test of the cross-over study was executed on the test data of dataset B (13 patients, 76 images). The external test was executed on all of dataset A (52 patients, 57 images).

### 3.1. Internal Test of PCL Segmentation on the EUS Images

We performed an internal test to evaluate the deep learning-based PCL segmentation on the EUS images. The models were developed by dataset A (39 patients, 43 images). The internal test data were collected from dataset A (13 patients, 14 images) not included in training data.

Table 2 shows the segmentation performance of the Basic U-Net, Residual U-Net, U-Net++, and Attention U-Net. The overall DSC and IoU scores of all deep-learning models were in a range of 0.727–0.794 and 0.628–0.741, respectively. In particular, the Attention U-Net showed the highest DSC and IoU scores (0.794 and 0.741, respectively); the U-Net++ showed the lowest performance, with a DSC of 0.727 and IoU of 0.628. The Attention U-Net had the highest pixel accuracy (0.983) and yielded the highest recall score at IoU > 0.70 and IoU > 0.85 (0.857 and 0.571, respectively).

**Figure 3:**
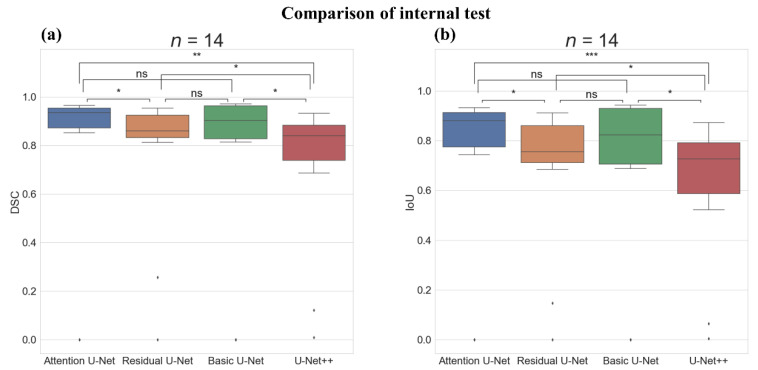
Comparison of the internal test with Boxplot representation and Wilcoxon test results between the deep-learning algorithms. (**a**) Boxplot representation and Wilcoxon test results of dice similarity score (DSC) and (**b**) Boxplot representation, and Wilcoxon test results of intersection over union score (IoU). ns: not significant, *: *p*-value < 0.05, **: *p*-value < 0.01, ***: *p*-value < 0.001, Number of images (*n*) = 14.

Figure 3 shows the boxplot and Wilcoxon test results of the Basic U-Net, Residual U-Net, U-Net++, and Attention U-Net models on the internal test. There was a statistically significant difference in the DSC score between the Attention U-Net and Residual U-Net (*p*-value < 0.05) and between the Attention U-Net and U-Net++ (*p*-value < 0.01); however, there was no statistically significant difference between the Attention U-Net and Basic U-Net. The IoU score showed a statistically significant difference between the Attention U-Net and Residual U-Net (*p*-value < 0.05) and between the Attention U-Net and U-Net++ (*p*-value < 0.001); however, there was no statistically significant difference between the Attention U-Net and Basic U-Net.

**Figure 4:**
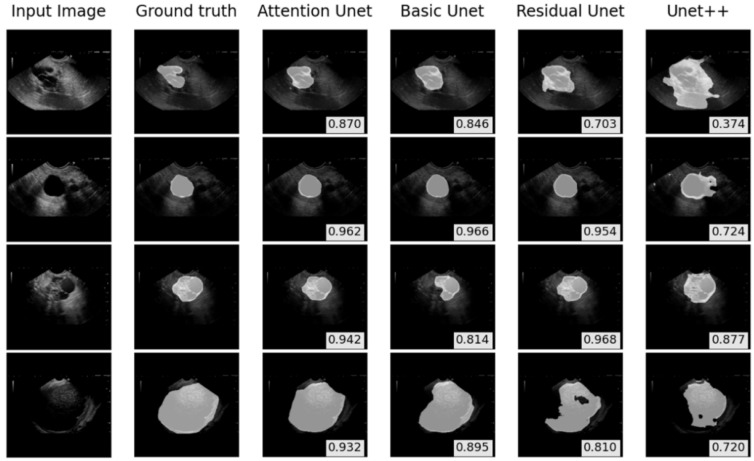
The performance of internal test on pancreatic cyst lesions segmentation. The dice similarity coefficient scores are shown in the bottom-right corners.

Figure 4 shows the comparisons between the ground truth and prediction using the deep learning-based models. The BCE–Dice loss was compared with the BCE loss and Dice loss by training the Attention U-Net. As shown in Table 3, the Attention U-Net models trained by the Dice loss showed the highest DSC and IoU scores (0.813 and 0.744, respectively). However, the trained model by the BCE-Dice loss showed the highest recall score at IoU > 0.85 (0.571).

**Figure 5:**
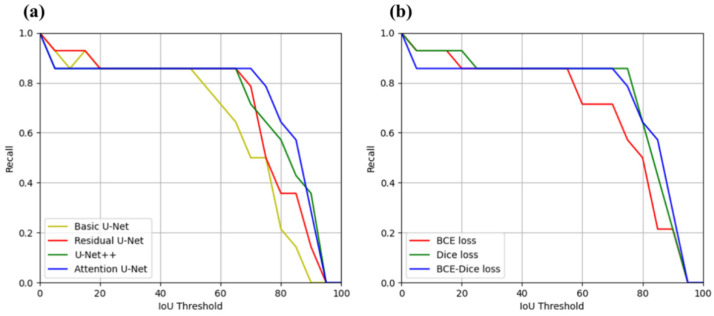
Recall–IoU curves: (**a**) Comparison of the four deep learning-based segmentation models and (**b**) Comparison of the three-loss functions on the Attention U-Net. IoU: intersection over union; BCE: binary cross-entropy.

Figure 5a shows the comparison of the deep-learning models on the Recall–IoU curve. The recall score of all models gradually decreased as the IoU threshold increased. However, the Attention U-Net performed better on the four deep-learning models. Figure 5b shows the comparison of the Attention U-Net models trained using the three-loss functions. The model trained by the BCE–Dice loss showed the highest recall score when the IoU score was greater than 0.8.

### 3.2. External Test of PCL Segmentation on the EUS Images

We performed an external test to evaluate the deep learning-based PCL segmentation on the EUS images. The models were developed by dataset A (39 patients, 43 images). The external test data was all of dataset B (59 patients, 364 images).

Table 4 shows the segmentation performance of models on the external test. The overall DSC and IoU scores of all deep-learning models were in a range of 0.614–0.703 and 0.488–0.595, respectively. The Basic U-Net showed the highest DSC, IoU scores, and recall at IoU > 0.70 (0.703, 0.595, and 0.437, respectively). The Attention U-Net showed the second-leading performance of DSC and IoU scores (0.691 and 0.587, respectively) and showed the highest recall score at IoU > 0.50 and IoU > 0.85 (0.709 and 0.176, respectively); however, the Residual U-Net showed the lowest performance, with a DSC of 0.614 and IoU of 0.488.

**Figure 6:**
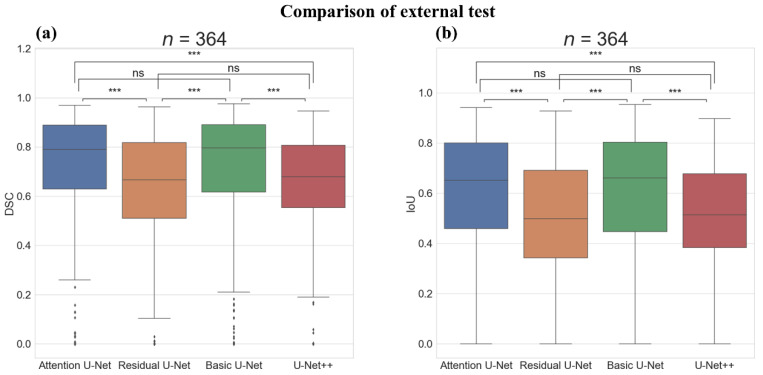
Comparison of external test with Boxplot representation and Wilcoxon test results between the deep-learning algorithms. (**a**) Boxplot representation and Wilcoxon test results of dice similarity score (DSC) and (**b**) Boxplot representation, and Wilcoxon test results of intersection over union score (IoU). ns: not significant, ***: *p*-value < 0.001, Number of images (*n*) = 364.

**Figure 7:**
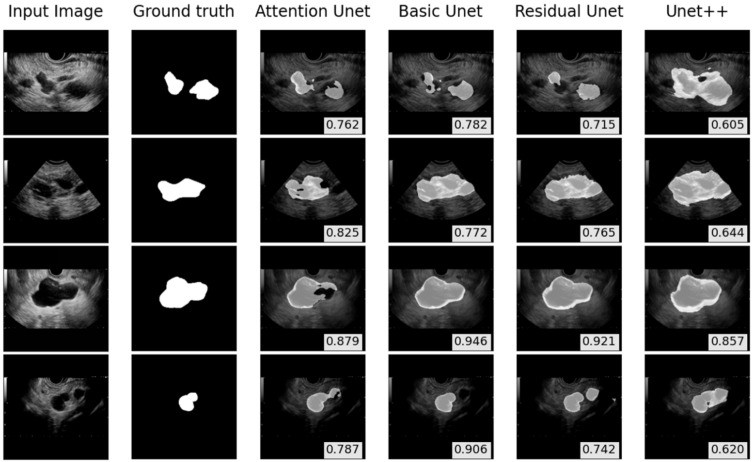
The performance of external test on pancreatic cyst lesions segmentation. The dice similarity coefficient scores are shown in the bottom-right corners.

Figure 6 shows the boxplot and Wilcoxon test results of the models on the external test. There was a significant difference in the DSC and IoU score between the Attention U-Net and Residual U-Net (*p*-value < 0.001) and between the Attention U-Net and U-Net++ (*p*-value < 0.001). Moreover, there was a significant difference between the Basic U-Net and Residual U-Net (*p*-value < 0.001) and between the Basic U-Net and U-Net++ (*p*-value < 0.001); however, there was no significant difference between the Attention U-Net and Basic U-Net. The comparison of segmentation results on the external test is illustrated in Figure 7.

### 3.3. Cross-Over Study of Dataset

The cross-over study was conducted in this study. We developed the models by using the training data of dataset B (46 patients, 288 images). Additonally, the internal test of the cross-over study was conducted by using the test data of dataset B (13 patients, 76 images) Next, the external test of cross-over study was executed by using all of dataset A (52 patients, 57 images).

#### 3.3.1. Internal Test on the Cross-Over Study

We performed an internal test of the cross-over study to evaluate the deep learning-based PCL segmentation on the EUS images. The models were developed by dataset B (46 patients, 288 images). The internal test data were collected from dataset B (13 patients, 76 images), not included in the training data.

Table 5 shows the segmentation performance on the internal test of the cross-over study. The overall DSC and IoU scores of all deep-learning models were in a range of 0.749–0.790 and 0.627–0.688, respectively. In particular, the Attention U-Net showed the highest DSC and IoU scores (0.790 and 0.688, respectively). The U-Net++ had the highest recall score at IoU > 0.85 (0.329).

**Figure 8:**
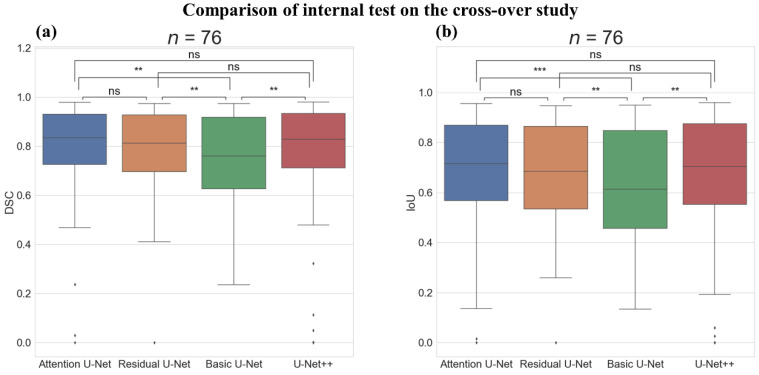
Comparison of the internal test on the cross-over study with Boxplot representation and Wilcoxon test results between the deep-learning algorithms. (**a**) Boxplot representation and Wilcoxon test results of dice similarity score (DSC) and (**b**) Boxplot representation, and Wilcoxon test results of intersection over union score (IoU). ns: not significant, **: *p*-value < 0.01, ***: *p*-value < 0.001, Number of images (*n*) = 76.

Figure 8 shows the boxplot and Wilcoxon test results on the internal test of the cross-over study. There was a statistically significant difference in the DSC score between the Attention U-Net and Basic U-Net (*p*-value < 0.01), between the Residual U-Net and Basic U-Net (*p*-value < 0.01), and between the U-Net++ and Basic U-Net (*p*-value < 0.01); however, there was no statistically significant difference between the Attention U-Net and Residual U-Net. Furthermore, there was no statistically significant difference between the Attention U-Net and U-Net++. The IoU score showed a statistically significant difference between the Attention U-Net and Basic U-Net (*p*-value < 0.001), between the Residual U-Net and Basic U-Net (*p*-value < 0.01), and between the U-Net++ and Basic U-Net (*p*-value < 0.01); however, there was no statistically significant difference between the Attention U-Net and Residual U-Net. Additionally, there was no statistically significant difference between the Attention U-Net and U-Net++.

#### 3.3.2. External Test on the Cross-Over Study

We performed an external test of the cross-over study to evaluate the deep learning-based PCL segmentation on the EUS images. The models were developed by dataset B (46 patients, 288 images). The external test data were all of dataset A (52 patients, 57 images).

Table 6 shows the segmentation performance on the external test of the cross-over study. The overall DSC and IoU scores of all deep-learning models were in a range of 0.660–0.691 and 0.565–0.583, respectively. The Basic U-Net showed the highest DSC and IoU scores (0.691 and 0.583, respectively). Additionally, the highest recall at IoU score > 0.50 was performed on the Basic U-Net (0.702). However, the highest recall at IoU score > 0.85 was performed on the U-Net++ (0.211).

**Figure 9:**
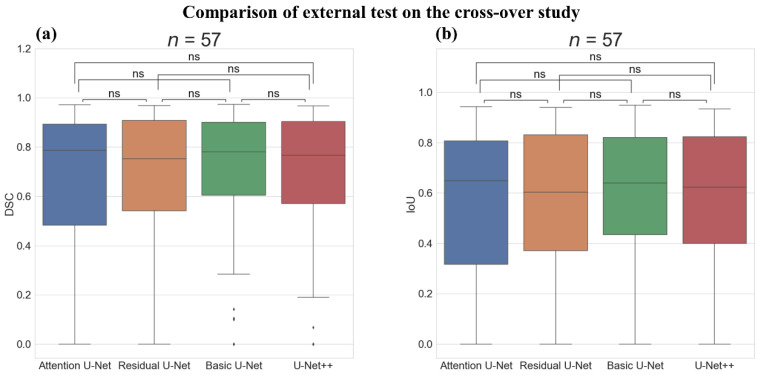
Comparison of the external test on the cross-over study with Boxplot representation and Wilcoxon test results between the deep-learning algorithms. (**a**) Boxplot representation and Wilcoxon test results of dice similarity score (DSC) and (**b**) Boxplot representation, and Wilcoxon test results of intersection over union score (IoU). ns: not significant, Number of images (*n*) = 57.

Figure 9 shows the boxplot and Wilcoxon test results of the models on the external test of the cross-over study. Both the DSC and IoU score of all models showed no statistically significant difference from each other.

## 4. Discussion

In this study, we implemented a deep learning-based automated algorithm for segmenting PCLs on EUS images.

We trained the models with dataset A for PCL segmentation. When evaluating the PCL segmentation on the internal test, the Attention U-Net yielded the highest results with respect to the DSC (0.794) and IoU (0.741). These results were better than those of the Basic U-Net, Residual U-Net, and U-Net++ models. Moreover, the Attention U-Net showed a statistically significant difference between the Residual U-Net and U-Net++, respectively.

The pixel accuracy and specificity of all models showed a high score (>0.960). This can be explained by the fact that the EUS images contain large areas of negative pixels that are not related to the PCLs. Although the Residual U-Net yielded the highest pixel sensitivity, the DSC and IoU scores of the Residual U-Net were lower than those of the Attention U-Net and Basic U-Net.

The Attention U-Net trained with BCE–Dice loss, which is a combination of the BCE and Dice loss, showed a higher recall score at IoU > 0.85 than models trained with only the BCE loss or Dice loss. Although the trained model with Dice loss showed the highest mean DSC and IoU score, the Attention U-Net yielded the highest recall score at a high IoU threshold. This result implied that the Attention U-Net made more segmented results showing a high IoU score.

On the external test, the Basic U-Net presented a higher DSC and IoU score than the Attention U-Net. However, there was no statistically significant difference between the Basic U-Net and Attention U-Net. Furthermore, the Attention U-Net showed the highest recall at IoU > 0.50 and IoU at > 0.75. Moreover, all deep-learning models on the external test yielded a lower segmentation performance than the internal test. However, the total external data (59 patients, 364 images) was bigger than the raw internal training data (39 patients, 43 images) used to build the models. Therefore, this result implied that the deep-learning algorithm could perform the PCL segmentation on a large amount of EUS images with a small raw training set. Moreover, deep-learning algorithms generally yield high performance with a large training set. Therefore, it is implied that the deep-learning algorithm could show better generalizability for PCL segmentation on EUS images with a large training set.

We also conducted a cross-over study that developed the models by using dataset B (46 patients, 288 images) and evaluated the models by using dataset B data (13 patients, 76 images) not included in the training data, and all of dataset A (52 patients, 57 images), respectively. On the internal test of the cross-over study, the Attention U-Net yield the highest DSC and IoU scores. Although the U-Net++ showed the highest recall score at IoU > 0.85, there was no significant difference between the Attention U-Net and U-Net++. On the external test of the cross-over study, the Basic U-Net performed the highest DSC and IoU score. The U-Net++ showed the highest recall score at IoU > 0.85. However, all of the models showed no statistically significant difference from each other. Moreover, the external test of the cross-over study showed a lower performance than the results of the internal test. We developed the models by using a large dataset (288 images) and evaluated the models by using a small dataset (57 images) on the external test. However, the large training dataset (288 images) was collected from a smaller number of patients (46 patients) than the external test data (52 patients). Although the total number of dataset B is 364 images, those are non-independent samples that were acquired from only 59 patients. This means that dataset B represents the data diversity of only 59 patients. Therefore, the models could learn the data diversity of only 39 patients in the training process. This implied that not only the number of images but also the number of patients are important for developing the deep learning models.

There are several limitations in the development of the PCL segmentation algorithm. First, the pixel properties of EUS images can be affected by hardware factors because there is a large difference among ultrasound images in their resolution, frequency, and depth [28,29]. In addition, ultrasound images have different brightness and contrast values, and they are often affected by speckle, shadows, and missing boundaries [30]. Second, the quality of EUS images can differ between EUS operators. The EUS procedure is highly EUS operator-dependent because it requires experienced techniques for endoscopic manipulation [30,31]. Third, there are various types of pancreatic cysts; therefore, the image can vary according to location, size, shape, and pattern. Applying digital signal processing and image processing techniques such as speckle suppression, stationary noise suppression, and window filtering during pre-processing or post-processing can improve the segmentation performance. Furthermore, the use of a large dataset would help to build a more generalized segmentation algorithm. Nonetheless, a deep learning-based PCL segmentation algorithm could assist with clinical diagnoses by automating the identification of PCLs on EUS images.

## Figures and Tables

**Table 1:** Dataset description.

Patient Characteristics	Dataset A	Dataset B
Number of Data		
Patients	52	59
Images	57	364
Gender, N(%)		
Male	20 (38.5)	36 (61.0)
Female	32 (61.5)	23 (39.0)
Age, N(%)		
25–44	5 (9.6)	4 (6.8)
45–64	18 (34.6)	17 (28.8)
65 and over	29 (55.8)	38 (64.4)

N: Number of patients.

**Table 2:** Performance comparison of the deep-learning models for pancreatic cyst lesion segmentation on the internal test. The mean of performance is shown with the standard deviation in parentheses on the accuracy, specificity, sensitivity, dice similarity coefficient, and intersection over union. A recall score greater than the threshold is shown.

Model	Accuracy	Specificity	Sensitivity	DSC	IoU	Recall at IoU > 0.50	Recall at IoU > 0.70	Recall at IoU > 0.85
Basic U-Net	0.980 (0.017)	0.990 (0.013)	0.774 (0.326)	0.780 (0.323)	0.719 (0.307)	0.857	0.714	0.429
Residual U-Net	0.976 (0.014)	0.984 (0.013)	0.857 (0.252)	0.775 (0.272)	0.691 (0.264)	0.857	0.786	0.357
U-Net++	0.962 (0.031)	0.970 (0.024)	0.799 (0.310)	0.727 (0.280)	0.628 (0.262)	0.857	0.500	0.143
Attention U-Net	0.983 (0.012)	0.991 (0.009)	0.797 (0.327)	0.794 (0.326)	0.741 (0.308)	0.857	0.857	0.571

DSC: Dice similarity coefficient; IoU: Intersection over union.

**Table 3:** Performance comparison of the loss functions of the Attention U-Net for pancreatic cyst lesion segmentation on the internal test. The mean of performance is shown with the standard deviation in parentheses on the accuracy, specificity, sensitivity, dice similarity coefficient, and intersection over union. A recall score greater than the threshold is shown.

Loss Function	DSC	IoU	Recall at IoU > 0.50	Recall at IoU > 0.70	Recall at IoU > 0.85
BCE Loss	0.770 (0.282)	0.689 (0.277)	0.857	0.714	0.214
Dice Loss	0.813 (0.268)	0.744 (0.266)	0.857	0.857	0.429
BCE-Dice Loss	0.794 (0.326)	0.741 (0.308)	0.857	0.857	0.571

BCE: Binary cross-entropy; DSC: Dice similarity coefficient; IoU: Intersection over union.

**Table 4:** Performance comparison of the deep-learning models for pancreatic cyst lesion segmentation on the external test. The mean of performance is shown with the standard deviation in parentheses on the accuracy, specificity, sensitivity, dice similarity coefficient, and intersection over union. A recall score greater than the threshold is shown.

Model	Accuracy	Specificity	Sensitivity	DSC	IoU	Recall at IoU > 0.50	Recall at IoU > 0.70	Recall at IoU > 0.85
Basic U-Net	0.971 (0.036)	0.986 (0.018)	0.759 (0.298)	0.703 (0.265)	0.595 (0.263)	0.687	0.437	0.168
Residual U-Net	0.957 (0.041)	0.970 (0.031)	0.761 (0.313)	0.614 (0.260)	0.488 (0.247)	0.495	0.239	0.041
U-Net++	0.956 (0.039)	0.966 (0.030)	0.817 (0.258)	0.640 (0.214)	0.503 (0.208)	0.514	0.190	0.005
Attention U-Net	0.972 (0.037)	0.989 (0.014)	0.723 (0.322)	0.691 (0.283)	0.587 (0.276)	0.709	0.434	0.176

DSC: Dice similarity coefficient; IoU: Intersection over union.

**Table 5:** Performance comparison of the deep-learning models for pancreatic cyst lesion segmentation on the internal test of the cross-over study. The mean of performance is shown with the standard deviation in parentheses on the accuracy, specificity, sensitivity, dice similarity coefficient, and intersection over union. A recall score greater than the threshold is shown.

Model	Accuracy	Specificity	Sensitivity	DSC	IoU	Recall at IoU > 0.50	Recall at IoU > 0.70	Recall at IoU > 0.85
Basic U-Net	0.872 (0.159)	0.973 (0.026)	0.965 (0.033)	0.749 (0.168)	0.627 (0.210)	0.658	0.638	0.250
Residual U-Net	0.877 (0.176)	0.979 (0.023)	0.972 (0.026)	0.781 (0.168)	0.668 (0.202)	0.829	0.474	0.276
U-Net++	0.811 (0.238)	0.984 (0.022)	0.973 (0.028)	0.768 (0.223)	0.665 (0.239)	0.789	0.513	0.329
Attention U-Net	0.864 (0.210)	0.982 (0.024)	0.973 (0.028)	0.790 (0.194)	0.688 (0.217)	0.829	0.539	0.316

DSC: Dice similarity coefficient; IoU: Intersection over union.

**Table 6:** Performance comparison of the deep-learning models for pancreatic cyst lesion segmentation on the external test of the cross-over study. The mean of performance is shown with the standard deviation in parentheses on the accuracy, specificity, sensitivity, dice similarity coefficient, and intersection over union. A recall score greater than the threshold is shown.

Model	Accuracy	Specificity	Sensitivity	DSC	IoU	Recall at IoU > 0.50	Recall at IoU > 0.70	Recall at IoU > 0.85
Basic U-Net	0.714 (0.287)	0.983 (0.030)	0.962 (0.038)	0.691 (0.271)	0.583 (0.272)	0.702	0.368	0.175
Residual U-Net	0.719 (0.287)	0.984 (0.026)	0.962 (0.038)	0.687 (0.263)	0.576 (0.272)	0.632	0.386	0.193
U-Net++	0.702 (0.300)	0.979 (0.037)	0.958 (0.044)	0.660 (0.299)	0.565 (0.299)	0.632	0.404	0.211
Attention U-Net	0.726 (0.298)	0.975 (0.044)	0.957 (0.048)	0.671 (0.295)	0.570 (0.297)	0.667	0.421	0.193

DSC: Dice similarity coefficient; IoU: Intersection over union.

## Data Availability

The datasets generated and/or analyzed during the current study are available from the corresponding author upon reasonable request.
